# Why Care: Complex Evolutionary History of Human Healthcare Networks

**DOI:** 10.3389/fpsyg.2020.00199

**Published:** 2020-02-13

**Authors:** Sharon E. Kessler

**Affiliations:** ^1^Department of Psychology, Faculty of Natural Sciences, University of Stirling, Stirling, United Kingdom; ^2^Department of Anthropology, Durham University, Durham, United Kingdom

**Keywords:** care-giving, illness, disease, social cognition, self-domestication, human evolution, niche construction, eusocial

## Abstract

One of the striking features of human social complexity is that we provide care to sick and contagious individuals, rather than avoiding them. Care-giving is a powerful strategy of disease control in human populations today; however, we are not the only species which provides care for the sick. Widespread reports occurring in distantly related species like cetaceans and insects suggest that the building blocks of care for the sick are older than the human lineage itself. This raises the question of what evolutionary processes drive the evolution of such care in animals, including humans. I synthesize data from the literature to evaluate the diversity of care-giving behaviors and conclude that across the animal kingdom there appear to be two distinct types of care-behaviors, both with separate evolutionary histories: (1) social care behaviors benefitting a sick individual by promoting healing and recovery and (2) community health behaviors that control pathogens in the environment and reduce transmission within the population. By synthesizing literature from psychology, anthropology, and biology, I develop a novel hypothesis (Hominin Pathogen Control Hypothesis) to explain how these two distinct sets of behaviors evolved independently then merged in the human lineage. The hypothesis suggests that social care evolved in association with offspring care systems whereas community health behaviors evolved as a type of niche construction. These two types of behaviors merged in humans to produce complex, multi-level healthcare networks in humans. Moreover, each type of care increases selection for the other, generating feedback loops that selected for increasing healthcare behaviors over time. Interestingly, domestication processes may have contributed to both social care and community health aspects of this process.

## What Are Healthcare Behaviors?

Human healthcare, including biomedical care, has enabled our species to exert an unprecedented amount of control over the pathogens which affect our species (Ferguson et al., 2003; Kessler et al., 2017, 2018). We synthesize medications, track the evolution and outbreak of novel diseases (Jones et al., 2008), and have even eradicated pathogens using vaccines (Ferguson et al., 2003). While these activities are clearly unique to our species, once healthcare behaviors are separated from medical technologies, we see intriguing continuities and convergences in healthcare behaviors across the animal kingdom. It is these patterns of care-giving behaviors that offer the opportunity to examine the underlying evolutionary processes driving them, including when, how, and why, care-giving in health contexts got scaled up in our species.

Here, I define healthcare behaviors as a group of behaviors which can control diseases. Because the outcomes of individual infections influence transmission dynamics within a community, this definition includes both behaviors that can control disease progression within infected individuals and behaviors that can control transmission through communities.

This definition means that healthcare behaviors can be paradoxical. Some behaviors promote recovery when given to diseased individuals, i.e., provisioning or guarding animals that might not survive otherwise, whereas other behaviors are harmful to diseased individuals, but protective for the community. For example, termites cannibalizing nest-mates which have been infected with a fungal pathogen do not benefit the infected individuals, but if the cannibalization deactivates the fungal propagules as they pass through the cannibalizer’s digestive tract, it does protect other nest-mates from exposure (Octavio Lopez-Riquelme and Luisa Fanjul-Moles, 2013). In addition, some healthcare behaviors, like grooming, are widespread across taxa (Lehmann et al., 2007; Octavio Lopez-Riquelme and Luisa Fanjul-Moles, 2013; Bush and Clayton, 2018), while others are restricted to only a few relevant species. For example, lifting a sick conspecific to breathe at the surface of a body of water (Bearzi et al., 2018) is only necessary in aquatic mammals. Understanding the complex transmission dynamics produced by different types of behaviors and their distribution across species is central to understanding how healthcare behaviors evolve.

In the next section, I examine healthcare behaviors observed in non-human animals while paying particular attention to who the beneficiary is (a specific individual vs. the community in general) and the implications of the behavior for disease transmission. I use *healthcare behaviors* as an umbrella term which encompasses both healthcare behaviors directed toward an individual [*social care*, hereafter, also referred to as conspecific care or social support in the anthropological literature (DeGusta, 2003; Hublin, 2009; Turner et al., 2014)] and healthcare behaviors which benefit the community (*community health behaviors*, hereafter, to reflect the division between medical care for patients and public health in humans today). I deal with these two types of healthcare behaviors separately because a central goal of this paper is to further our understanding of how these two processes may have interacted during human evolution. Notably, these definitions exclude self-directed care and other forms of care, like parental care, which are not specific to health contexts.

Because animals (including humans) may have difficulty determining when a conspecific has an infectious vs. non-infectious condition (injury, disability, non-contagious diseases, etc.), social care for individuals with infectious and non-infectious conditions are unlikely to have evolved independently. As it is more costly to provide care to infectious individuals (one might contract the disease), if animals are unable to accurately distinguish infectious from non-infectious individuals, yet still provide care, non-infectious individuals will lower the costs of providing care when *averaged over many care-giving events*. Over an evolutionary scale, this may be relevant to understanding how social care could be perpetuated in populations, despite the risks that they pose for the carer. Therefore, I include responses to non-contagious conditions as well. Similarly, I also include responses to dead individuals because (1) care behaviors may start before death and continue afterward (Anderson et al., 2010; Bearzi et al., 2018) and (2) corpses are potential sources of pathogens (Octavio Lopez-Riquelme and Luisa Fanjul-Moles, 2013; Cremer et al., 2018; Porter et al., 2019).

To my knowledge, this is the first review which integrates the biological literature (animal behavior, citations below) with the psychological and anthropological literature [e.g., compassion (Gilbert, 2017; Seppälä et al., 2017) and attachment theory (Fogel and Melson, 1986; Preston, 2013; Cassidy and Shaver, 2016), fossil evidence of social care during human evolution (Dettwyler, 1991; Lebel et al., 2001; DeGusta, 2003; Hublin, 2009; Spikins, 2015; Spikins et al., 2018, 2019)] to produce a new hypothesis explaining the integration of social care and community health behaviors. These healthcare behaviors are part of the behavioral immune system (Schaller and Park, 2011) but focus on a specific subset of the behavioral immune system – the contexts in which individuals suppress disgust, fear, and avoidance responses to engage in behaviors that benefit others (Peng et al., 2013; Preston, 2013). While this review aims to provide a broad overview, I pay particular attention to primates for their evolutionary relationship to humans, cetaceans, and birds for their convergences with humans in cognition, and eusocial insects (Hymenoptera: ants, bees, wasps, and Isoptera: termites) for their convergences with humans without complex cognition. While the selection of species may appear an unsystematic collection of anecdotes, this is largely a reflection of the discipline at present; these are the taxa which have received the most attention, first as anecdotal reports by field researchers and then, with taxa specific reviews (e.g., Bearzi et al., 2018; Bush and Clayton, 2018; Reggente et al., 2018; Watson and Matsuzawa, 2018). This has had the unintended effect of making the discipline fairly “siloed.” One of the goals of this paper is to look across taxa and behaviors to identify patterns and start building a broader theoretical framework for understanding the evolution of healthcare behaviors. I hope that this will lay the foundation for future work which can test this theoretical framework.

## Social Care: Behaviors Benefitting an Infected Individual

### Grooming

Allogrooming is widespread across the animal kingdom [i.e., primates (Lehmann et al., 2007), birds (Bush and Clayton, 2018), ungulates (Hart and Hart, 2018), and insects (Octavio Lopez-Riquelme and Luisa Fanjul-Moles, 2013)]. This includes removing ectoparasites with hands (Lehmann et al., 2007), bills (Bush and Clayton, 2018), teeth (Hart and Hart, 2018), or mouthparts (Octavio Lopez-Riquelme and Luisa Fanjul-Moles, 2013). Although many species appear to tolerate some ectoparasites (Hart, 2011), they may also be vectors of other diseases (Sadanandane et al., 2018), making their removal beneficial for both the parasitized individual and the wider social group. Analyses of grooming patterns have shown that for many species, grooming is a key mechanism for establishing and maintaining relationships with kin, allies, or mates and maintaining group cohesion (Lehmann et al., 2007). However, it also serves important hygienic functions (Hart and Hart, 2018). While most animals can self-groom, social grooming is particularly important for areas of the body which the animal cannot reach (Hart and Hart, 2018). For example, allopreening occurs in at least 50 families of birds and controls parasites on the head and neck areas that the bird itself can’t reach (Bush and Clayton, 2018). Fungal infections are a key driver of allogrooming in many eusocial insects (Octavio Lopez-Riquelme and Luisa Fanjul-Moles, 2013; Cremer et al., 2018; Liu et al., 2019). If the infectious spores aren’t removed before they penetrate the cuticle, they cause an infection which is fatal to the infected individual and dangerous to other nest-mates (Octavio Lopez-Riquelme and Luisa Fanjul-Moles, 2013; Cremer et al., 2018; Liu et al., 2019). These examples demonstrate the importance of allogrooming to both in the infected individual and to the broader community (Octavio Lopez-Riquelme and Luisa Fanjul-Moles, 2013; Cremer et al., 2018; Liu et al., 2019).

Another component of grooming is wound-cleaning, which may include removing debris and licking wounds, behavior which manually washes debris out of a wound and applies saliva to it (Hart, 2011). Saliva has antibacterial properties which may promote healing (Hart and Powell, 1990; Hart and Hart, 2018). For example, termite-hunting ants (*Megaponera analis*), which incur high levels of injuries when hunting, carry wounded nest-mates back to the nest and provide care to the injuries (Frank et al., 2018). Ants with one or two bitten off legs are carried back and the wounds are licked by the other ants (Frank et al., 2018). Recovery of ants provided with such care is 80%, compared to 10% in ants who received no care (Frank et al., 2018). Ants who have healed after having extremities bitten off are able to return to hunting, potentially explaining why this species evolved this form of social care (Frank et al., 2018). Interestingly, ants who had five legs removed are not carried back to the nest, but this appears to be regulated by the injured ant itself, in that it is unable to position itself correctly to be carried back (Frank et al., 2018).

The extent to which grooming may increase transmission through exposing the groomer is unknown and likely depends on the transmissibility of the parasite and how intimately the groomer interacts with the infected individual. However, because grooming often occurs along established social (often kinship) networks, it is possible that because these individuals are already likely to be in close proximity that grooming does not significantly elevate the risk that already exists (Griffin and Nunn, 2012).

### Social Anointing

Self-anointing occurs when individuals rub substances, or even ants, on their bodies (Bush and Clayton, 2018). Social anointing occurs when individuals apply it to others (Bowler et al., 2015). These behaviors are common in eusocial insects which secrete antifungal and antibacterial substances which they apply to nest-mates through allogrooming (Octavio Lopez-Riquelme and Luisa Fanjul-Moles, 2013; Liu et al., 2019). In eusocial Hymenoptera, these substances are secreted by Dufour’s, mandibular, venom, and metapleural glands and in Isoptera by sternal glands, head glands, and rectal fluids (Octavio Lopez-Riquelme and Luisa Fanjul-Moles, 2013). Many insect species also secrete antimicrobial substances and apply them to their eggs or larvae in the nest (Octavio Lopez-Riquelme and Luisa Fanjul-Moles, 2013).

While self-anointing, including with ants, occurs frequently in birds (Bush and Clayton, 2018), to my knowledge, only one bird species has been observed socially anointing. The crested auklet (*Aethia cristatella*), a colonial species of seabird, anoints prospective mates with a substance released from its specialized wick-like feathers (Douglas, 2008). The volatile compounds from the secretions paralyze and kill lice (Douglas, 2013).

Within primates, social anointing appears to be restricted to a few new world monkey taxa: the untufted capuchins (Alfaro et al., 2012) (*Cebus*), tufted capuchins (Alfaro et al., 2012) (*Sapajus*), and owl monkeys (Jefferson et al., 2014) (*Aotus*). Monkeys have been observed socially anointing using a variety of strongly smelling plants, mud, or insects which can be crushed into the fur or stimulated into secreting compounds (millipedes, stink bugs, ants) (Alfaro et al., 2012). Interestingly, in general *Cebus* tends to use more plants, while *Sapajus* uses more insects (Alfaro et al., 2012). Most social anointing occurs with group-mates in physical contact, but still directing most (or all) of the rubbing to their own bodies (Alfaro et al., 2012). However, anointing others, particularly infants, has also been observed and, even when most rubbing is self-directed, being in physical contact with others who are anointing helps to distribute the substances more evenly (Alfaro et al., 2012). Analyses of which body parts get covered has shown that self-anointing is focused on areas that are out of sight on the body, while social anointing tends to increase coverage of areas that are hard to reach, suggesting that social anointing does have a hygienic effect (Bowler et al., 2015). One of the leading hypotheses for social-anointing in general is that it is mutual medication (Bowler et al., 2015), which serves to protect individuals against ectoparasites and biting flies, both through individual protection and by reducing the general attractiveness of the group to parasites.

In many mammals, grooming may be performed with the mouth (i.e., tongue, teeth, etc.), meaning that it involves applying saliva to another individual (Hart and Hart, 2018). The extent to which this serves as social anointing is currently unknown, because it’s unclear to what extent the saliva protects against pathogens when applied to fur or an uninjured body surface. This would be an interesting area for future work.

### Guarding

Remaining near or guarding (repelling others) can protect a vulnerable individual from attacks by conspecifics and predators. It has been observed in chimpanzees [*Pan troglodytes* (Anderson, 2016; Watts, 2019)], gorillas [*Gorilla beringei* (Porter et al., 2019; Watts, 2019)], marmosets [*Callithrix jacchus* (Bezerra et al., 2014)], ring-tailed lemurs [*Lemur catta* (Nakamichi et al., 1996)], snub-nosed monkeys [*Rhinopithecus roxellana* (Yang et al., 2016)], elephants [*Loxodonta africana* (Douglas-Hamilton et al., 2006)], giraffe [*Giraffa camelopardalis* (Bercovitch, 2013; Strauss and Muller, 2013)], peccaries [*Pecari tajacu* (de Kort et al., 2018)], dingos [*Canis dingo* (Appleby et al., 2013)], mongooses [*Helogale parvula* (Rasa, 1983)], and pinnipeds (Reggente et al., 2018). This behavior includes waiting for a conspecific that cannot keep up, standing over a conspecific that is unable to move, or chasing away conspecifics and predators (citations above). In general, guarding is frequently given to kin or to past or future mates [marmosets (Bezerra et al., 2014), gorillas (Porter et al., 2019), and snub-nosed monkeys (Yang et al., 2016)]. For species at a high risk of predation or intra-species aggression, guarding is likely to be valuable. This investment is likely to be costly for the carer, as it may require the carer to forgo foraging opportunities, incur higher predation risks when separated from the social group and standing near a vulnerable individual who may attract predators, or engage in aggressive encounters when driving away others (Bercovitch, 2013; Strauss and Muller, 2013). The extent to which the carer incurs a risk of disease transmission will depend on the proximity of the carer to the contagious individual and how transmissible the pathogen is (Porter et al., 2019). When the individual is infectious, driving away others may also decrease exposure within the population (Hart and Hart, 2018).

### Provisioning

Provisioning wounded or ill group members has been observed in wild mongooses [*H. parvula* (Rasa, 1983)], lions [*Panthera leo* (Hart, 2011)], foxes (Hart, 2011), and giant otters [*Pteronura brasiliensis* (Davenport, 2010)]. This may take the form of tolerating a food theft or providing food to a begging individual (Davenport, 2010). Interestingly, these species may share food and/or cooperatively raise young. For cooperatively breeding species, such care may be a form of kin selection (Rasa, 1983). If the injured/sick individual is related to the carer and may help rear future offspring to whom the carer will also be related, providing care is an investment in both the injured/sick individual and in the future offspring (Rasa, 1983). Provisioning is likely to be particularly valuable to individuals undergoing a long period of injury or illness that prevents obtaining their own food (Sugiyama, 2004; Davenport, 2010). The costs to the carer will likely depend on whether the carer is still able to obtain adequate nutrition. The extent to which the carer incurs a risk of disease transmission will depend on the proximity of the carer to the contagious individual and how transmissible the pathogen is.

### Carrying/Supporting

Carrying and/or supporting a sick or injured individual (hereafter, carrying) has been observed in multiple species (e.g., Appleby et al., 2013; Reggente et al., 2018), but received particular attention in two taxa: non-human primates (Watson and Matsuzawa, 2018) and cetaceans (Bearzi et al., 2018). In primates mothers (and others) may carrying infants long after death (Watson and Matsuzawa, 2018), even as the corpses putrefy and decay (Biro et al., 2010). Corpse carrying has been observed in chimpanzees, bonobos (*P. paniscus*), orangutans (*Pongo abelii*), gorillas, multiple species of macaques (*Macaca* ssp.), geladas (*Theropithecus gelada*), langurs (*Rhinopithecus bieti*), snub nosed monkeys and capuchins (Watson and Matsuzawa, 2018). One wild chimpanzee mother was observed carrying the body of her infant for over 2 months after it died of a respiratory illness (Biro et al., 2010). During that time, the body swelled and mummified (Biro et al., 2010). For primate and non-primate species that do not carry, physical constraints, like body size, may make it impossible (Nakamichi et al., 1996). For example, ringtail lemur mothers have been observed attempting to carrying older infants that were unable to move on their own, but too big for the mother carry (Nakamichi et al., 1996).

However, within species that do carry dead infants, we also do not yet have a good understanding of the variation that we observe in carrying behavior (Watson and Matsuzawa, 2018). We do not yet understand why, even within the same population or social group, some females carry and others do not (Watson and Matsuzawa, 2018). Similarly, we also do not understand why, even within the same female, she may carry one of her infants when it dies but not another (Watson and Matsuzawa, 2018). She may even engage in both carrying behavior and cannibalizing the corpse (Watson and Matsuzawa, 2018). The behavior appears to be complex and maybe influenced by the age and weight of the infant, how the infant died, the social rank and experience of the mother, and the climate (Watson and Matsuzawa, 2018).

The other taxa in which carrying behavior has received particular attention are the aquatic mammals – species in which carrying and lifting an animal to the surface to breathe can be life-saving (Bearzi et al., 2018). In cetaceans attending to a corpse has been observed to continue for up to a week (Bearzi et al., 2018). In an analysis of reports of attentiveness to dead conspecifics across cetaceans, 20 of 88 living species were found to engage in it (*N* = 78 records) (Bearzi et al., 2018). However, dolphins accounted for 92% of these records (Bearzi et al., 2018). Of the cases where the sexes of the potential carers were known (*N* = 28), 75% included adult females and an immature who may have been the females’ offspring (Bearzi et al., 2018). While this appears to suggest that maternal bonds may be frequent conduits for care-giving in the intelligent and socially complex dolphins, the authors point out that there are several factors that make it difficult to generalize with confidence (Bearzi et al., 2018). They include unequal amounts of research effort across species, differences in dive behavior across species influencing the likelihood of observing a species at the surface, and differences in corpse buoyancy across species (more likely in species with thick blubber) and within species (influenced by gaseous build up during decomposition and possibly by age/size) (Bearzi et al., 2018). These factors may make the sample biased (Bearzi et al., 2018).

### Thermoregulatory Assistance

Thermoregulatory assistance has not received a great deal of attention across species, but is an interesting avenue for future work. Honeybees (*Apis mellifera*) produce a behavioral fever in the brood-comb when larvae become infected with the heat-sensitive fungus *Ascosphaera apis* (Starks et al., 2000). The bees isometrically contract their thoracic muscles to raise their thoracic temperature and put them near the brood cells (Wilson-Rich et al., 2009). A study with an experimental infection showed that infected colonies raised the brood-comb temperature above the pre-infection temperature (Starks et al., 2000). No such increase was observed in the control colony (Starks et al., 2000). While other species, particularly birds and reptiles, may control pathogens by sunning (Bush and Clayton, 2018), those behaviors are generally self-directed, rather than providing care to others. Researchers working with species that huddle or engage in torpor, may want to look for instances of inducing behavioral fevers in conspecifics who are ill.

## Community Health Behaviors

### Nest Sanitation

Nest sanitation behaviors like removing waste and replacing contaminated/infested nest materials are common in birds (Ibáñez-Álamo et al., 2014; Ibanez-Alamo et al., 2016; Diego Ibanez-Alamo et al., 2017; Bush and Clayton, 2018) and eusocial insects (Octavio Lopez-Riquelme and Luisa Fanjul-Moles, 2013). For example, house wrens (*Troglodytes aedon*) are thought to reduce the abundance of mites (*Dermanyssus*) by removing old nesting material and great tits (*Parus major*), blue tits (*Cyanistes caeruleus*), and pied fly catchers (*Ficedula hypoleuca*) show more nest sanitation behaviors when ectoparasites are present than when they are not (Bush and Clayton, 2018). Phylogenetic analyses of parental nest sanitation showed that parental removal of nestling feces drove the evolution of fecal sacs (a mucus covering that encloses nestling feces and accompanying bacteria) (Ibáñez-Álamo et al., 2014; Diego Ibanez-Alamo et al., 2017). Moreover, experimental studies showed that breaking the fecal sacs resulted in nestlings with more ectoparasites and lower probabilities of survival (Azcarate-Garcia et al., 2019), suggesting that feces removal is beneficial for nestling health.

Eusocial insects also show nest sanitation behaviors, particularly for waste removal (Octavio Lopez-Riquelme and Luisa Fanjul-Moles, 2013). Honeybees defecate when flying away from the nest, paper wasps which build nests that hang from trees drop larval meconial outside the nests, and other taxa (ants, aphids, social mites, and others) defecate in refuse dumps located away from the nest, at the border of the nest, or in special chambers within the nest (Octavio Lopez-Riquelme and Luisa Fanjul-Moles, 2013). These behaviors reduce the risk of nests transmitting infections to nest-mates (Octavio Lopez-Riquelme and Luisa Fanjul-Moles, 2013). A promising area of future research would be to conduct comparative work on other nest-living, burrowing, and den-living taxa.

Interesting comparisons in non-nest living species also include the evolution of latrine behaviors, in which animals defecate in restricted areas. There are extensive literatures on animal latrines focusing on intraspecific studies of the communicative functions (e.g., Irwin et al., 2004; Barja and List, 2006; Wronski et al., 2006; Jordan et al., 2007; Ruibal et al., 2010; Droescher and Kappeler, 2014; Rodgers et al., 2015; Barocas et al., 2016; Eppley et al., 2016; King et al., 2017) and seed dispersal (Feeley, 2005; Pouvelle et al., 2009; Dos Santos Neves et al., 2010; Gonzalez-Zamora et al., 2012; Zarate et al., 2019), but the distribution of defecation behaviors across species, their implications for concentrations of pathogens across the landscape (Nunn and Dokey, 2006; Nunn et al., 2011, 2014; Numberger et al., 2019), and how this may drive the evolution of latrine behaviors is not fully understood.

### Nest Fumigation

This behavior shows interesting convergences between birds and insects. Several bird species, most commonly cavity nesting birds, incorporate fresh aromatic herbs into their nests (Scott-Baumann and Morgan, 2015). The leading, non-mutually exclusive hypotheses are that it evolved through sexual selection (i.e., male starlings bringing herbs to nests as mating effort), nest protection hypothesis (herbs decrease parasites or pathogens, i.e., lice, mites, fleas, blowflies, midges, blackflies, or bacterial colony numbers, richness or diversity in nests), or the drug hypothesis (herbs do not reduce parasite numbers, but improve the health of the chicks directly, possibly by potentiating their immune systems) (Scott-Baumann and Morgan, 2015). Overall the evidence for these three hypotheses is suggestive of complex evolutionary causes, but not conclusively understood (Scott-Baumann and Morgan, 2015). The evidence for sexual selection is strongest in starlings where males bring the herbs until the females begin laying, and (in spotless starlings, *Sturnus unicolor*) the females may even remove the herbs as they are brought (Scott-Baumann and Morgan, 2015), suggesting that it is not left in the nest to benefit future offspring. In blue tits females bring the herbs during the hatchling period (Scott-Baumann and Morgan, 2015). Experimental manipulations of herbs in nests produced evidence suggesting that herbs decrease nest parasites/bacteria or increases in chick health/nest success, but usually did not produce simultaneous evidence of both (Scott-Baumann and Morgan, 2015). Similarly, house sparrows (*Passer domesticus*) and house finches (*Carpodacus mexicanus*) incorporate fibers of cigarette buts into their nests, with nests with a high density of them having lower mite densities (Bush and Clayton, 2018). Although less well studied, similar studies have linked green vegetation in songbirds with lower botfly infestations (*Philonis* spp.), pine materials with less blowfly larvae (*Protocalliphora*) in eagle nests (*Hieraaetus fasciatus*), and yarrow (*Achillea millefolium*) with fewer fleas in tree swallow nests (*Tachycineta bicolor*) (Bush and Clayton, 2018).

Similarly, insects also incorporate protective substances into their nests. For example, honeybees build their combs out of a mixture of resins that they have gathered and antibiotic substances in their saliva (Octavio Lopez-Riquelme and Luisa Fanjul-Moles, 2013). They also coat the walls of their nests with bodily secretions containing antimicrobials (Octavio Lopez-Riquelme and Luisa Fanjul-Moles, 2013). Termites construct their nests using soil and feces, which contain antimicrobial and antifungal substances (Octavio Lopez-Riquelme and Luisa Fanjul-Moles, 2013). Ants also secrete antimicrobial substances which they distribute on themselves, their nest-mates via allogrooming, and the nest (Octavio Lopez-Riquelme and Luisa Fanjul-Moles, 2013).

Nest fumigation with volatile compounds has been suggested to occur in insects Formosan subterranean termites [*Coptotermes formosanus* (Chen et al., 1998)] and red imported fire ants, [*Solenopsis invicta* (Wang et al., 2015)]. Nest fumigation in mammals has received less research, but there is some evidence for convergent evolution. The dusky-footed wood rat (*Neotoma fuscipes*) has also been documented to use bay leaves (*Umbellularia californica*), similarly to birds, to control fleas in the nest (Hemmes et al., 2002). Similar hypotheses have been suggested for the cedar, *Thuja occidentalis*, that flying squirrels, *Glaucomys sabrinus*, and red squirrels, *Tamiasciurus hudsonicus*, use to construct their nests (Patterson et al., 2007). Additional research into the choice of nesting materials in small mammals would be particularly informative in understanding the evolution of fumigation behaviors. Overall, the diversity of ways in which anti-pathogenic substances are incorporated into nests across taxa suggest that there are strong selective pressures for reducing pathogens in nests.

### Undertaking: Disposal of the Dead (Burial, Removal, Cannibalism)

Corpse management appears to be relatively unusual outside of humans and insects. Rodents will bury corpses in response to olfactory cues emitted through decomposition (Pinel et al., 1981) and in one case, a wolf mother (*Canis lupus*) was inferred to have buried her dead pups (Boyd et al., 1993). The researchers found locations where pups appeared to have been buried, then subsequently dug up and taken away by scavengers (Boyd et al., 1993). The authors speculated that the mother may have buried the first few pups after they died while still caring for the remaining pups until they too died (Boyd et al., 1993). The cause of death was thought to be canine distemper virus or canine parvovirus (Boyd et al., 1993).

Eusocial insects show an intriguing diversity of corpse management strategies, including combinations of necrophoresis (transporting the dead), necrophagy/cannibalism (eating dead, injured, or diseased individuals), burial, or necrophobia (avoidance) (Octavio Lopez-Riquelme and Luisa Fanjul-Moles, 2013; Sun and Zhou, 2013). There are two broad combinations which vary by taxa (Hymenoptera and Isoptera) (Octavio Lopez-Riquelme and Luisa Fanjul-Moles, 2013). Eusocial Hymenoptera: In these taxa, the primary strategy is necrophoresis, but the brood may be eaten (Octavio Lopez-Riquelme and Luisa Fanjul-Moles, 2013). Corpses, refuse piles, and locations where corpses were generally avoided (Octavio Lopez-Riquelme and Luisa Fanjul-Moles, 2013). Burial is not a primary strategy, but does occur (Octavio Lopez-Riquelme and Luisa Fanjul-Moles, 2013). In Isoptera necrophagy is the main strategy, but corpses are also buried (Octavio Lopez-Riquelme and Luisa Fanjul-Moles, 2013). Corpses and burial locations are generally avoided (Octavio Lopez-Riquelme and Luisa Fanjul-Moles, 2013). Because corpses are sources of disease, disposing of them is both an important way of reducing pathogens in the nest and a dangerous activity for the individuals dealing with the corpses (Octavio Lopez-Riquelme and Luisa Fanjul-Moles, 2013).

### Undertakers: Divisions of Labor

Eusocial insects are well known for their complex divisions of labor within the colony, and in some taxa, this includes undertaking and hygienic behaviors (Octavio Lopez-Riquelme and Luisa Fanjul-Moles, 2013; Stroeymeyt et al., 2014). In the eusocial Hymenoptera, the main strategy of corpse management is corpse removal, and this task is performed primarily by subcastes of workers who specialize in these tasks (Octavio Lopez-Riquelme and Luisa Fanjul-Moles, 2013; Sun and Zhou, 2013). These individuals are frequently older, with genetic, hormonal, and neurological differences from others which may predispose them to being sensitive to the chemical signals of death and working without a circadian rhythm enabling quick removal of corpses (Octavio Lopez-Riquelme and Luisa Fanjul-Moles, 2013). This specialization, particularly by age, means that younger individuals tend to work inside the nest and tend to the brood, while older individuals engage in riskier tasks outside the nest which bring them in to contact with additional pathogens (Octavio Lopez-Riquelme and Luisa Fanjul-Moles, 2013; Stroeymeyt et al., 2014). In colonies of fungus growing ants, there is a strict division of labor among workers who forage and workers who transport waste to garbage dumps (Octavio Lopez-Riquelme and Luisa Fanjul-Moles, 2013; Stroeymeyt et al., 2014). The dump workers are older ants who are actively rejected by other nest-mates if they try to leave the dump, thus enforcing strict spatial and social barriers to pathogen transmission (Octavio Lopez-Riquelme and Luisa Fanjul-Moles, 2013; Stroeymeyt et al., 2014).

In contrast, Isoptera do not have a subcaste of workers which specializes in corpse disposal (Octavio Lopez-Riquelme and Luisa Fanjul-Moles, 2013; Sun and Zhou, 2013). Instead, corpses are generally eaten or buried and burials are generally performed by groups of workers (Octavio Lopez-Riquelme and Luisa Fanjul-Moles, 2013). While termite workers may not specialize in undertaking, in some species soldiers do not participate in burials (Octavio Lopez-Riquelme and Luisa Fanjul-Moles, 2013). While I have discussed the broad patterns that we observe, these behaviors are complex and do vary between species within the Hymenoptera and Isoptera (Octavio Lopez-Riquelme and Luisa Fanjul-Moles, 2013). Engaging in these risky behaviors like waste and corpse removal is likely to be maintained by kin selection (Cremer et al., 2007; Octavio Lopez-Riquelme and Luisa Fanjul-Moles, 2013; Sun and Zhou, 2013; Stroeymeyt et al., 2014; Shakhar, 2019). In eusocial insects, the workers are non-reproductive, but highly related to the others in the colony, thus performing tasks that benefit the colony as whole, including the reproductives, is a way for them to pass a portion of their genes in to future generations (Cremer et al., 2007; Octavio Lopez-Riquelme and Luisa Fanjul-Moles, 2013; Sun and Zhou, 2013; Stroeymeyt et al., 2014; Shakhar, 2019).

## The Hominin Pathogen Control Hypothesis

**FIGURE 1:**
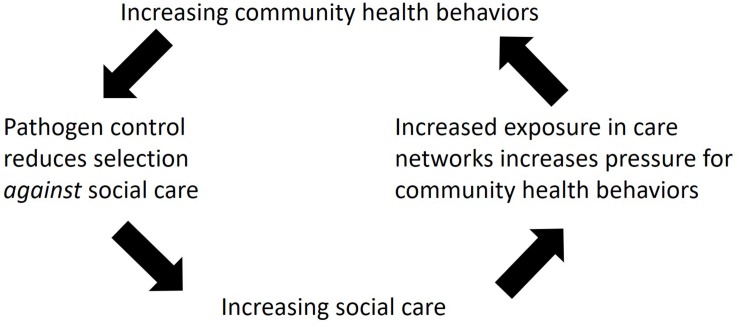
Schematic diagram of the Hominin Pathogen Control Hypothesis. The arrows show the hypothesized selection pressures predicted to increase social care and community health behaviors in hominins during human evolution.

When we look across animals, the two types of healthcare behaviors, social care and community heath behaviors, produce a mosaic pattern across species. This section of the paper proposes a novel, testable hypothesis (Hominin Pathogen Control Hypothesis, Figure 1) which explains the evolution and integration of these two types of healthcare behaviors in humans, based on the patterns we see across animals. The hypothesis suggests that social care evolved in association with offspring care systems and social cognition pathways in the brain (Preston, 2013). Thus, many of the social care behaviors that are common between humans and animals, i.e., guarding sick individuals, are likely shared with our most recent common ancestors. In contrast, the evolutionary history of community health behaviors appears to be different. Using niche construction theory (Laland et al., 2016, 2017) as a framework for understanding community health behaviors (Hurtado, personal communication, 2018) enables these behaviors to be understood as techniques for controlling pathogens in the constructed environment, e.g., within nests. This interpretation suggests that many of the community health behaviors common between humans and animals, i.e., birds or insects, are derived behaviors that evolved through convergent evolution.

Finally, one of the striking characteristics of human healthcare behaviors is how frequent and widespread they are. The Hominin Pathogen Control Hypothesis predicts that feedback loops created by social care and community health behaviors created increasing pressure on each type of behavior (Figure 1). Social care, when given to sick and contagious individuals, is predicted to actually *increase* the risk of disease transmission to susceptible carers, thereby putting the broader community at risk (Kessler et al., 2018). This is predicted to create selection for community health behaviors that reduce transmission (e.g., sanitation, fumigation, disposal of the dead), thereby *reducing* selection *against* social care and allowing it to become more frequent in the population. Overall, this produces a feedback loop that selects for increasing social care and community health behaviors over time (Figure 1).

### Social Care Uses Parent–Offspring Care and Social Cognition Pathways

Many of the behaviors categorized as social care are behaviors that are commonly given from parents to vulnerable offspring, e.g., provisioning and carrying. Moreover, carers are frequently, although not always, mothers. And indeed, one of the leading hypotheses explaining behaviors like continuing to carry around corpses long after they have started to decompose, providing strong visual, olfactory, behavioral, and tactile cues of death, is that it maybe a by-product of the maternal bond (Biro et al., 2010; Bearzi et al., 2018; Watson and Matsuzawa, 2018).

Social care, when given to a genetic relative, may increase the carer’s inclusive fitness if the recipient recovers and reproduces (Kessler et al., 2017, 2018; Shakhar and Shakhar, 2015; Shakhar, 2019). The idea that inclusive fitness may be a key driver of care among relatives is also supported by the frequent appearance of species which tend to show cooperative behaviors [i.e., alliances in primates and cetaceans (Chapais, 1995; Parsons et al., 2003), and cooperative hunting/provisioning of young in some carnivores (Davenport, 2010)].

Potential links between offspring care and social care for the sick have also attracted attention from researchers focused on the proximate mechanisms of compassion (Gilbert, 2017; Seppälä et al., 2017) and attachment (Fogel and Melson, 1986; Cassidy and Shaver, 2016). Preston (2013) unifies ultimate explanations of altruistic responses to distressed or needy individuals with the proximate mechanisms underlying offspring care systems. Altruistic responding is defined as, “as any form of helping that applies when the giver is motivated to assist a specific target after perceiving their distress or need (Preston, 2013, p. 1307).” Social care for the sick fits well within that definition and can be thought of as a subtype of altruistic responding. Preston (2013) roots the mechanisms of altruistic responding in the physiology and neurobiology of offspring care systems, describing the role of oxytocin in reducing avoidance behaviors, dopamine in motivating approach behaviors, and the anterior cingulate cortex and prefrontal cortex in regulating emotion and decision-making processes.

However, while the cues that sick individuals provide may overlap with those of offspring (i.e., inability to forage), they are not the same. This suggests that the process for recognizing when individuals need care requires more than simply activating offspring care behaviors. There is a growing consensus that the process of evaluating the health status of others is an aspect of social cognition (Fisher et al., 2014; Shakhar and Shakhar, 2015; Steinkopf, 2015; Tiokhin, 2016; Kessler et al., 2017; Kavaliers and Choleris, 2018; Steinkopf and de Barra, 2018). The same brain pathways that enable animals to interpret behavioral, olfactory, vocal, or visual cues to discern the identities, motivations, and intentions of others can likely detect health cues such as lethargy or difficulties moving, odor changes due to immune responses, respiratory infections in vocalizations, or fevers and rashes on faces (Fisher et al., 2014; Shakhar and Shakhar, 2015; Steinkopf, 2015; Tiokhin, 2016; Kessler et al., 2017; Kavaliers and Choleris, 2018; Steinkopf and de Barra, 2018). This would suggest that recognizing health cues in others may be a key aspect of social cognition and/or that these pathways may have been co-opted for that use (Fisher et al., 2014; Shakhar and Shakhar, 2015; Steinkopf, 2015; Tiokhin, 2016; Kessler et al., 2017; Kavaliers and Choleris, 2018; Steinkopf and de Barra, 2018).

This is *tentatively* supported by the frequent observations of care in species with greater cognitive abilities and complex social relationships like the cetaceans (Bearzi et al., 2018), primates (Anderson et al., 2010), or carnivores (Davenport, 2010). It may suggest that the aspects of the social cognition which facilitate close relationships may contribute to the development of care-relationships (Fisher et al., 2014; Shakhar and Shakhar, 2015; Steinkopf, 2015; Tiokhin, 2016; Kessler et al., 2017; Kavaliers and Choleris, 2018; Steinkopf and de Barra, 2018). However, this observation must, at present, remain tentative because they are also taxa which have received a great deal of research effort *because of* their reputations for social complexity. There is a need for investigations into understudied species and for researchers to report a *lack of social care* when opportunities were present but no care was given. Turner et al. (2014) is an excellent example of this, reporting that a population of Japanese macaques (*Macaca fuscata*) with a high rate of congenital limb malformations, does not provide care to disabled group members.

### Community Health Behaviors: Pathogen Control as a Key Element of Niche Construction

All living organisms modify their environment (Laland et al., 2017). This includes striking behaviors like building webs, nests, or dams, and more subtle environmental changes like plants altering the temperature, moisture level, and composition of the soil around them (Laland et al., 2017). This phenomenon in which organisms modify, or “construct,” aspects of their environment is called niche construction (Laland et al., 2017).

Niche construction theory (Laland et al., 2016, 2017) can be used as a framework for understanding how community health behaviors can control pathogens in the constructed environment (Hurtado, personal communication, 2018). As pathogen control techniques, they are part of the species behavioral immune system [psychological and behavioral defenses against disease (Schaller and Park, 2011)] and contribute to the social immunity of the population [collective defenses against disease (Cremer et al., 2007; Stroeymeyt et al., 2014)].

Nests, in particular, are likely to be extreme examples due to the elevated risks of disease transmission in densely populated, enclosed environments (Cremer et al., 2007; Octavio Lopez-Riquelme and Luisa Fanjul-Moles, 2013). This may make selection to control disease transmission particularly strong on nest-building species. Similarly, the enclosed, controlled environment of a nest may offer more opportunities to construct it in ways that reduce the success of pathogen transmission (Octavio Lopez-Riquelme and Luisa Fanjul-Moles, 2013; Laland et al., 2017). This may include controlling air quality through fumigations or modifications to alter airflow, reducing energetic costs for those in the nest through protection from precipitation, wind, and temperature extremes (Octavio Lopez-Riquelme and Luisa Fanjul-Moles, 2013; Laland et al., 2017). For animals which live in cold environments, huddling behaviors can increase nest temperatures. Similarly, shaded nests may reduce energy costs in hot climates. These energy savings may enable individuals to invest more in immune defenses. At the same time, fumigations, building the nest with materials that have anti-parasitic properties, cleaning and anointing others, and removing waste and corpses likely reduce the quantity and diversity of pathogens (Octavio Lopez-Riquelme and Luisa Fanjul-Moles, 2013; Scott-Baumann and Morgan, 2015), effectively modifying the distributions of pathogens in the environment.

While nests are particularly visible examples, other species construct their environments as well. This includes changing the distributions of prey species through predation, distributing seeds and parasite larvae as they defecate, etc. (Laland et al., 2016, 2017). Animal behavior produces selection on their pathogens to adapt to environmental changes the animals bring about and this, in turn, generates selection on the host species, producing feedback loops (Laland et al., 2016, 2017). During human evolution, these feedback loops may have reduced selection pressure *against* social care, enabling increasing social care over time and greater pressure for pathogens to be controlled via community health behaviors (Figure 1).

### Social Care in the Fossil Record

There are numerous fossil hominins which have been determined to have suffered from severe illnesses and disabilities, including Shanidar I with a severely damaged right arm and Aubesier 11 with severe tooth loss (Dettwyler, 1991; Lebel et al., 2001; DeGusta, 2003; Hublin, 2009; Spikins et al., 2019). This has led to vigorous debates about whether fossil evidence is sufficient to infer social care. When researchers have suggested that various conditions were so debilitating that it would have been impossible for the individual in question to survive without care, primatologists have frequently rebutted these arguments with evidence that wild primates survive similar afflictions without care. This has included primate populations where individuals recover from limbs being maimed or severed in snares (Byrne and Stokes, 2002; Munn, 2006; Stokes and Byrne, 2006; Beamish and O’Riain, 2014) and nearly complete tooth loss (Cuozzo and Sauther, 2004). However, while our understanding of wild primates’ resilience makes it difficult to argue that these hominins definitely received care (Dettwyler, 1991; Byrne and Stokes, 2002; DeGusta, 2002, 2003; Cuozzo and Sauther, 2004; Munn, 2006; Stokes and Byrne, 2006; Beamish and O’Riain, 2014; Turner et al., 2014), our knowledge of the types of care-giving provided by primates (and indeed more distant taxa, citations above) make it equally difficult to argue that hominins definitely did not provide care (Lebel et al., 2001; Hublin, 2009; Spikins, 2015; Spikins et al., 2018, 2019). While we do know that sometime between diverging from our last common ancestor with chimpanzees and today, hominins scaled up the care we give to others, we still cannot say exactly when that occurred.

We also still do not know how and when hominins began exhibiting increasing community health behaviors; however, the Hominin Pathogen Control Hypothesis (Figure 1) predicts that after social care and community health behaviors appeared in the human lineage, they should have been interdependent and increased together.

### Did Domestication Play a Role in the Evolution of Human Care?

Domestication itself does not appear to produce care-giving behaviors, in that when a species is domesticated, it does not appear to start providing care that the wild counterpart did not. However, the processes that humans underwent as we domesticated other species (Zeder, 2016, 2017), and possibly also ourselves (Hare, 2017), may have contributed to our cognitive predisposition to provide care and engage in extensive niche construction (Zeder, 2016, 2017).

Humans not only domesticated other species, but we have also been argued to have domesticated ourselves (Hare et al., 2012; Hare, 2017). We show some of the classic signs of domestication, including increased cooperation, communication, tolerance, prosociality, extended juvenile periods, and pedomorphic features (Hare et al., 2012; Hare, 2017; Benitez-Burraco and Kempe, 2018). Today 70–90% of care-giving is given within family networks (Kleinman, 1978). Since human social cognition is specialized for recognizing subtle changes in those we know well, we are particularly well positioned to notice when family members are ill (Kessler et al., 2017). We may hear respiratory infections in voice changes, see rashes or flushing from fever on faces, notice lethargy or signs of pain during movement, or blood shot eyes around in our white scleras (Provine et al., 2011; Fisher et al., 2014). Recent studies (Shakhar and Shakhar, 2015; Steinkopf, 2015; Tiokhin, 2016; Shakhar, 2019) have put forward provocative hypotheses suggesting that human symptoms are signals that evolved to influence care-giving and avoidance behaviors of others. This would be an exciting avenue for future research into if and how humans may be specialized for soliciting and providing social care. Thus, while human social cognition may have increased our ability to detect when those around us need care, the emotional and psychological changes associated with self-domestication (Hare et al., 2012; Hare, 2017) and cooperatively raising young (sensu Hawkes et al., 1998; Burkart et al., 2009; Hrdy, 2009; Tomasello, 2014) may have increased the likelihood that we would provide that care.

Domestication has been argued to be a type of niche construction (Zeder, 2016). While humans began domesticating plants and animals in the Neolithic, evidence suggests that we were dramatically altering the distribution of species on the landscapes on which we lived, going back to the disappearances of large megafauna in the Late Pleistocene (Boivin et al., 2016). In doing so, we likely altered the communities of pathogens that depended upon these species as well (Boivin et al., 2016). If so, it would suggest that human niche construction was extensive enough that it may have influenced pathogen communities long before agriculture and the breeding of domestic livestock in the Neolithic (Boivin et al., 2016).

Humans began domesticating other species in the Neolithic and since then, we have domesticated vast numbers of plants and animals (Boivin et al., 2016). We have dramatically altered and constructed our ecological niche, changing both the distributions of target species (prey species which became livestock, wild crops which became agriculture crops, and “pest” species) (Boivin et al., 2016). In doing so, we altered the biodiversity of pathogen communities that the domesticates evolved with, including diseases that can be zoonotic to humans, vector species attracted to livestock, commensal species like mice, etc. (Boivin et al., 2016). This meant that we likely re-engineered the distribution of pathogens in our environments, including creating high densities of our domesticated species and living at higher densities ourselves (Boivin et al., 2016). This probably created selection for controlling pathogens that spread through human populations or species on which we depended. In addition, humans also began constructing and living in shelters which likely created selection for disease control and “nest hygiene” in human communities, similarly to in birds and insects.

At this point, it is not possible to tease apart exactly what changes in human evolution produced the healthcare behaviors we see in humans today. Instead of being one causal factor, it seems more plausible that when we look across animals there are a number of things that increase the likelihood of healthcare behaviors evolving: extensive niche construction (Boivin et al., 2016), expanded kin networks and extended juvenile period (Hawkes et al., 1998; Burkart et al., 2009; Hill et al., 2009; Hrdy, 2009), cooperative behaviors (Tomasello, 2014), and increased cognition and communication (Benitez-Burraco and Kempe, 2018; Benitez-Burraco et al., 2018). Thus, human care-giving may reflect the integration of two distinct types of healthcare behaviors each with its own evolutionary history (1) selection to provide social care to those in our social networks and (2) selection to construct our niches in ways that facilitate the control of pathogens.

Interestingly, work on the evolution of eusociality in insects suggests that the evolution of social immunity behaviors may have been a prerequisite to the evolution of the high density eusociality seen today (Meunier, 2015). This raises the possibility that the same may have been true in humans – that social care and community health behaviors may have enabled later increases in human density and social complexity (Kessler et al., 2018).

### Summary and Future Directions

This paper provided a novel synthesis of animal care-giving in sickness contexts. I reviewed both social care behaviors which are directed at the sick individual and community health behaviors which benefit the community by controlling pathogens in the environment. In examining the mosaic of behaviors present across species, it appears that social care may have evolved in association with offspring care systems while community health behaviors may have evolved convergently in several taxa that engage in striking niche construction behaviors, like nest building. Finally, I introduced a novel hypothesis, the Hominin Pathogen Control Hypothesis, which predicts that human healthcare evolved through the integration of social care and community health behaviors. Aspects of this hypothesis could be tested in several ways:

(i) Test whether levels of social care and levels of community health behaviors covary, such that higher levels of one type of healthcare behavior should be associated with higher levels of the other. This could be tested across populations, i.e., nests within a species, or across species. Note that it would not be necessary for the same individuals who engage in social care to also engage in community health behaviors. The two types of healthcare behaviors could be carried out by different individuals. However, if the two types of healthcare behaviors do covary across populations or species, it would support the idea that they are linked.
(ii) Examine whether other taxa known for elaborate niche construction, also engage in community health behaviors.

This would support the idea that community health behaviors are a form of niche construction.
(iii) Conduct a comparative study using researcher surveys of when opportunities for social care were present and no care was given. A more systematic understanding of which species provide social care and how often would enable a quantitative analysis of how social care may overlap with different infant rearing systems. These results could support the idea that social care is rooted in offspring care systems.

## Author Contributions

SK wrote the manuscript and agreed to be accountable for the content of the work.

## Conflict of Interest

The authors declare that the research was conducted in the absence of any commercial or financial relationships that could be construed as a potential conflict of interest.
